# Regional free cash flow dataset: An approach to regional performance evaluation

**DOI:** 10.1016/j.dib.2019.104175

**Published:** 2019-06-21

**Authors:** Vladimir Kolmakov, Alexandra Polyakova

**Affiliations:** a Plekhanov Russian University of Economics, 36 Stremyanny Lane, Moscow, 117997, Russia; b Industrial University of Tyumen, 38 Volodarskogo Street, Tyumen, 625000, Russia

**Keywords:** Regional performance, Economic development, Free cash flow, Economic value, Regional economy

## Abstract

This data article provides estimates on the Russian regions' aggregate free cash flow, which is not covered by national statistics of major countries. A proper microeconomic model was adapted to regional level data to derive a synthetic indicator of a regional economy's performance. The data contributes to the set of regional performance measures thus enabling a new look at studies of economic growth and development. Conventional economic growth indicators, such as GDP, fixed capital investment or industrial output, are widely criticized since they can have negative values only in terms of growth rates thus showing no evidence of value creation or deterioration. Our data on regional free cash flow eliminates this drawback.

**Table:** Specifications table

Subject	Economics, Econometrics and Finance (General)
Specific subject area	Aggregate performance of a territory, economic development
Type of data	Table, Figure
How data were acquired	The data were acquired through the microeconomic model adaptation to regional level data. Calculations were made using publicly available statistics of the basic indicators (factors)
Data format	Raw
Parameters for data collection	The following conditions were considered for data collection: - Consistent time series – same length, missing values mean-substituted; - Factor tables were brought to uniform structure and sequence of observations (regions); - Factors' values were rescaled to uniform measure (thousands of rubles)
Description for data collection	Our estimates of the Russian regions' free cash flow are based on eight factors combined in a computation model. Basic factors' data are open access through the Federal Statistics Authority of Russia
Data source location	Russian Federation, 85 regions of the Russian Federation
Data accessibility	Data is with this article

**Table:** 

**Value of the data** • contemporary economic growth models demand an indicator of absolute measure of economic degrade or progress on the contrast to conventional performance indicators that mislead the research due to their “always positive” scale [1]. There is much evidence of “growth without development” and “development without growth” cases that require alternative data to be used in assessment [2]; • since conventional growth and development indicators are widely criticized [3], the indicator proposed is a proper substitute that enables the value-based approach implementation in economic systems' performance and development assessment; • the data represent the net worth of selected items of balance sheet statements, profit and loss statements and cash flow statements of the real sector economy aggregates broken down by regions to show a distinct value increment; • the data can assist in regional performance reassessment and in revision of economic growth and development models to provide a proper ground for value-based policies; • the data enable specific types of mathematical functions to employ both positive and negative absolute values; • the data sheet can be used as the template for pro-forma calculations of free cash flow of other economic systems given the necessary factor data is available.

## 1 Data

This article is associated to a Microsoft Excel Worksheet as a supplementary material. The data contain time series of free cash flow values on the 85 Russian regions covering the period of 2006–2016. No transformation was applied, except for scaling (all in thousands of Russian rubles). Missing values (<0.1% of total quantity of levels) were mean-substituted.

The data file has the “Model description” sheet containing the formalization of data retrieving procedure, the “0-FCF” sheet with the free cash flow indicator values calculated for each of the 85 regions through 2006–2016, sourced by eight factor-values sheets according to the methodology in use.

Factor data were retrieved from the Federal Statistics Authority of Russia online repository (http://fedstat.ru), formatted by us to the uniform structure, length and sequence of observations (regions). Appropriate links to indicators’ webpages are provided on respective data sheets.

## 2 Experimental design, materials, and methods

### 2.1 Data collection procedure

Proper foundations of the free cash flow use and different approaches to its calculation regarding the enterprise level are described in Lehn and Poulsen [4], Richardson [5], and Opler and Titman [6]. In line with the literature, we denote the free cash flow as the total worth of funds, generated by an economic system over a period, available to be withdrawn by all the types of capital providers “without harming a firm's ability to operate and to produce future cash flows” [7].

Then, the regional free cash flow is the net worth of an increment to economic potential of enterprises localized within a territory of a given region. Economic potential in this case is treated as enterprises' ability to maintain sustainable growth or to increase individuals’ wealth directly (through shareholder value) or indirectly (through the increase of tax revenue or labor demand) [8].

We calculate the regional free cash flow indicator using the following standard model [9] widely employed at enterprise-level valuations – see formula 1 and Table 1 for the indicator breakdown:

(1) $$\text{FC}{\text{F}}_{\text{i}\text{,}\text{j}}\;=\;\text{(}{\text{R}}_{\text{i}\text{,}\text{j}}-{\text{C}}_{\text{i}\text{,}\text{j}}\text{)}\text{(}1-\text{T)}\;+\;{\text{A}}_{\text{i}\text{,}\text{j}}-\text{F}{\text{I}}_{\text{i}\text{,}\text{j}}\;+\;\text{F}{\text{S}}_{\text{i}\text{,}\text{j}}-\text{((A}{\text{R}}_{\text{i}\text{,}\text{j}}\;+\;\text{S}{\text{I}}_{\text{i}\text{,}\text{j}}-\text{A}{\text{P}}_{\text{i}\text{,}\text{j}}\text{)}-\text{(A}{\text{R}}_{\text{i}\text{,}\text{j}-1}\;+\;\text{S}{\text{I}}_{\text{i}\text{,}\text{j}-1}-\text{A}{\text{P}}_{\text{i}\text{,}\text{j}-1}\text{))}$$

Where *i* stands for a region, *i*∈[1; 85].

**Table 1:** Free cash flow model breakdown.

Factor reference	Factor	Tab name in XLSX data file
FCF	Free cash flow	"0-FCF"
R	Sales revenue	"1-Sales revenue"
C	Cost of goods sold	"2-COGS"
A	Fixed assets depreciation and amortization	"3-D&A"
FI	Fixed capital investment	"4-CAPEX"
FS	Receipts from fixed assets sales	"5-Fixed assets sales"
AR	Accounts receivable from operations	"6-Trade receivables"
SI	Inventory book value	"7-Inventory"
AP	Accounts payable from operations	"8-Trade payables"
T	Corporate tax rate = 20%	–

*j* stands for a year, *j*∈[2006; 2016].

Since we are unable to reliably determine a proportion of cross-regional operations of an enterprise, we assume that all the operations are localized in a region of incorporation.

### 2.2 Data use and interpretation rule

Positive free cash flow indicates an increase in value, while the negative means value deterioration, as follows from the cash flow-based methodologies described in Oded and Allen [10]. Yet, a negative free cash flow value in a specific period does not necessarily mean that a regional property complex is used inefficiently: capital expenditure undermines free cash flow directly but has a potentially positive lagged effect. On the other hand, permanently negative free cash flow together with positive operating profit can hardly be an anticipated characteristic of economic development of a region since income reinvestment rate is going to be 100% and above, meaning the increase of debt, thus limiting the possibility to increase quality of life and individuals’ wealth in the long-term perspective. The same remark is applicable to permanently positive values of free cash flow, if they are maintained by investment downturn.

**Fig. 1:**
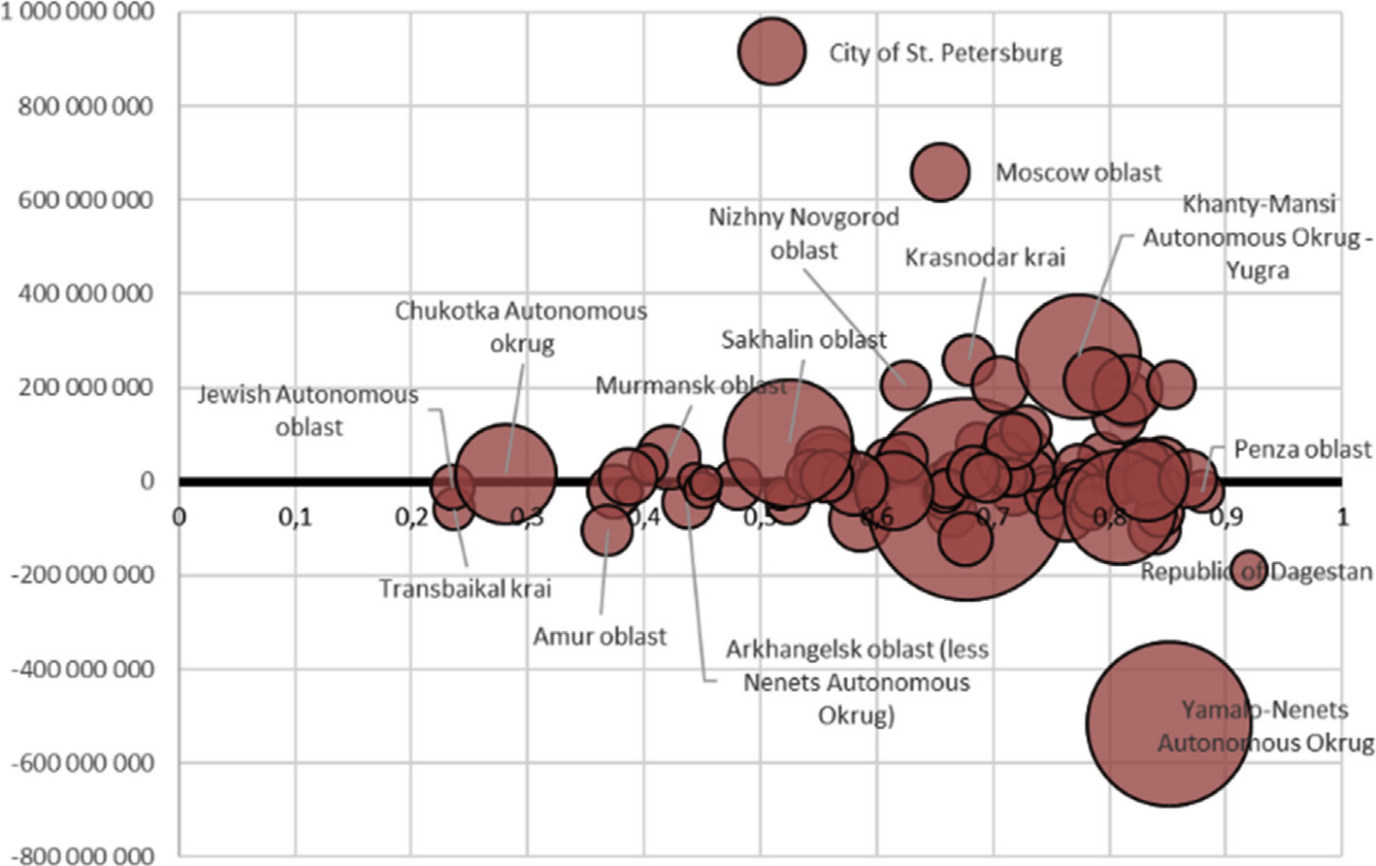
Free cash flow of the Russian regions depending on private sector share in total capital expenditure (GRP per capita used for diameters) in 2016.

The dataset with supplementary factors – GDP per capita and private sector share in total capital expenditure broken down by regions – allows to verify standard economic growth models from the value point of view. Combining the indicator with the private sector share in 2016-total capital expenditure gives a proper look at the data possible use (Fig. 1).

**Fig. 2:**
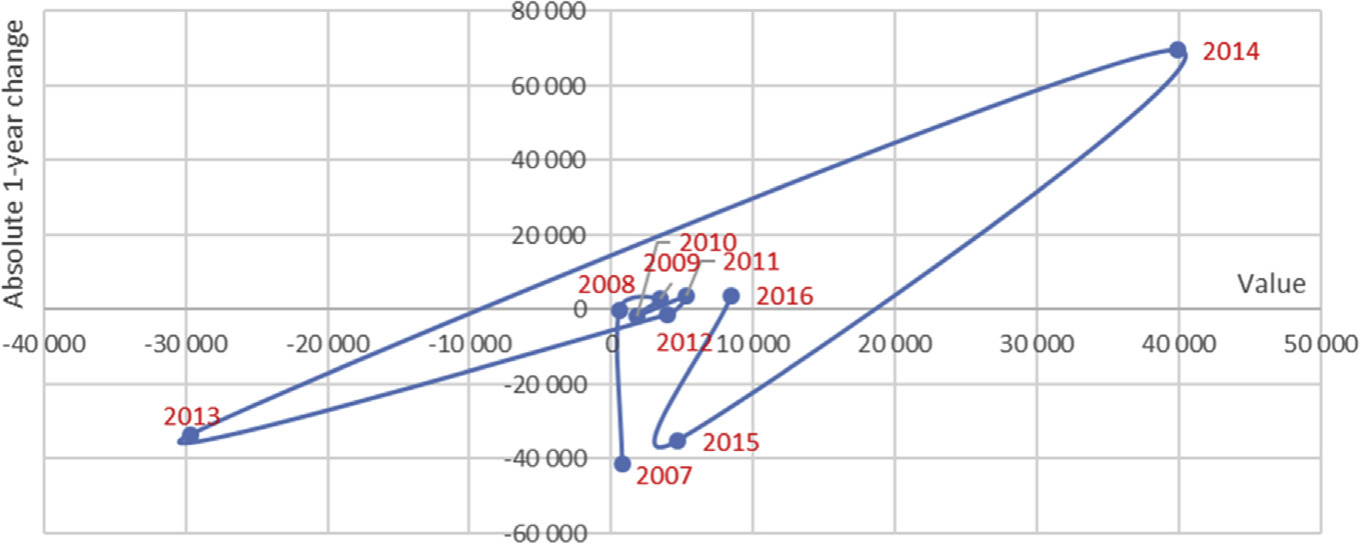
Aggregate free cash flow tracer-chart, all regions, 2016.

Thus, the data allow to reclassify regions according to their contribution to value creation and to detect cycles in individual and aggregate dynamics. Another possible use of the data is to trace a value growth pattern and to determine the development cycle of a region or of the whole economy (Fig. 2).

The tracer-chart indicates moderate value creation in 2008–2012 followed by significant volatility afterwards in terms of absolute changes. Matching with GDP or GRP dynamics can give an alternative view on growth and development.

Finally, the data can be decomposed into net operating profit and the net investment to estimate the two broad factors’ contribution to economic performance of a region, in response to Oguz and Knight proposition [11].
